# Monocytic meningitis complicating histiocytosis and response to MEK-inhibitor: a case series

**DOI:** 10.1007/s00277-025-06317-x

**Published:** 2025-03-25

**Authors:** Tom Abrassart, Ahmed Idbaih, Damien Roos-Weil, Damien Galanaud, Fleur Cohen-Aubart, Jean-François Emile, Pierre Boncoeur, Zahir Amoura, Danielle Seilhean, Julien Haroche, Matthias Papo

**Affiliations:** 1 Service de médecine interne 2, Centre de référence des histiocytoses, Sorbonne Université, Assistance Publique–Hôpitaux de Paris, Hôpital Pitié-Salpêtrière, Paris, France; 2 Service d’hématologie clinique, Hôpital Universitaire de Bruxelles, Université Libre de Bruxelles, Bruxelles, Belgique; 3 Service de neurologie, Sorbonne Université, Assistance Publique–Hôpitaux de Paris, Hôpital Pitié- Salpêtrière, Paris, France; 4 Sorbonne Université, Service d’hématologie clinique, Assistance Publique–Hôpitaux de Paris, Hôpital Pitié-Salpêtrière, Paris, France; 5 Service d’imagerie médicale, Sorbonne Université, Assistance Publique–Hôpitaux de Paris, Hôpital Pitié-Salpêtrière, Paris, France; 6 Paris-Saclay University, Versailles SQY University, Assistance Publique–Hôpitaux de Paris (AP-HP), Ambroise-Paré Hospital, Smart Imaging, Service de Pathologie, Boulogne, EA4340-BECCOH France; 7 Service d’hématologie biologique, Sorbonne Université, Assistance Publique–Hôpitaux de Paris, Hôpital Pitié-Salpêtrière, Paris, France; 8 Service de neuropathologie, Sorbonne Université Assistance Publique–Hôpitaux de Paris, Hôpital Pitié- Salpêtrière, Paris, France

**Keywords:** Monocytic meningitis, Monocytic pleocytosis, Erdheim-Chester disease, Histiocytosis

## Abstract

Central nervous system (CNS) involvement is common in histiocytosis, yet cerebrospinal fluid (CSF) analysis often yields normal results. We present three cases of monocytic meningitis associated with histiocytosis. The first patient was diagnosed with Erdheim-Chester disease (ECD) and exhibited evidence of a MAP2K1 mutation, concomitant with chronic myelomonocytic leukemia. Brain magnetic resonance imaging (MRI) revealed leptomeningitis and pachymeningitis. The presence of the same MAP2K1 mutation in CSF monocytes confirmed the clonal origin of neuromeningeal infiltration. Treatment with binimetinib rapidly improved the patient’s clinical condition. The second case involved CNS primary malignant histiocytosis (CNS-PMH) associated with myelodysplastic syndrome. However, treatment with binimetinib only led to a partial and time-limited response. The last patient was diagnosed with mixed histiocytosis ECD/Rosai-Dorfman disease (RDD). Cobimetinib also proved effective in managing CNS symptoms. CSF pleocytosis in CNS involvement of histiocytosis has been reported in a few published cases with neurological involvement. Given its rarity, the presence of monocytic meningitis should prompt immediate suspicion of histiocytosis, particularly if accompanied by typical manifestations. In cases of neurological involvement in histiocytosis, lumbar puncture and liquid biopsy can sometime overcome the need for a meningeal biopsy. The molecular characterization of histiocytosis is essential for considering the use of targeted therapy, but the lack of an identified mutation should not preclude the use of anti-MEK therapy.

## Introduction

 Histiocytoses (Erdheim-Chester disease (ECD), Langerhans-cell histiocytosis (LCH), Rosai-Dorfman disease (RDD) and malignant histiocytosis (MH)) are a group of diseases with diverse clinical presentations [1]. Central nervous system (CNS) involvement is common, but cerebrospinal fluid (CSF) analysis often yields normal results. However, pleocytosis associated with neurological involvement, predominantly lymphocytic and more rarely monocytic, has been reported in a few published cases. We present here three patients with monocytic meningitis associated with histiocytosis, and a review of the literature on meningitis in histiocytosis. We also describe the evolution of meningeal infiltration following the introduction of a MEK-inhibitor.

## Case presentation

### Case 1

**Fig. 1:**
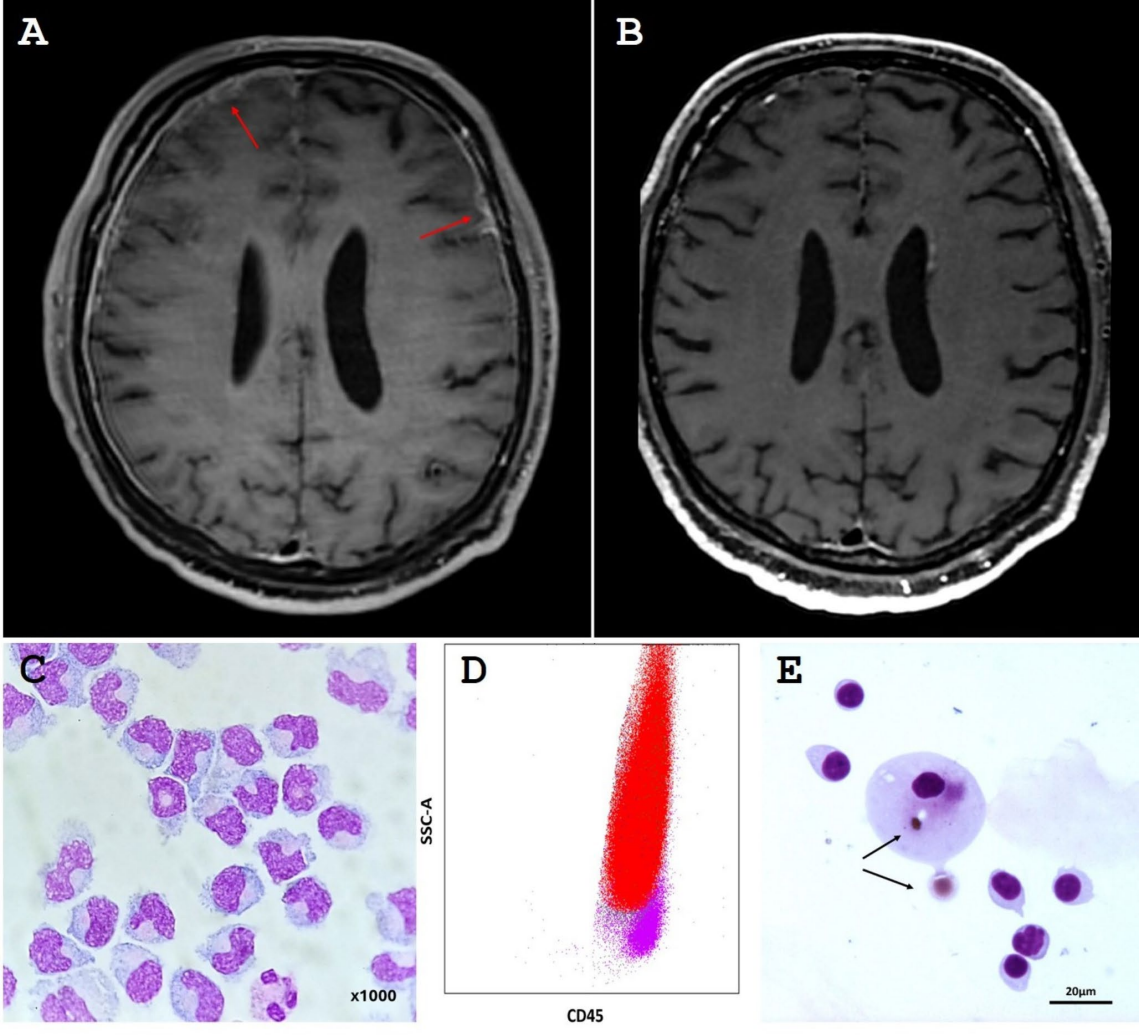
(**A-B**) Neurological involvement of Erdheim-Chester disease (case 1). Brain MRI revealed diffuse pachymeningitis (arrows) before treatment with binimetinib (**A**). Improvement of pachymeningitis 3 months after initiation of binimetinib (**B**). (**C-D**) Cerebrospinal fluid (CSF) of patient with central nervous system primary malignant histiocytosis (case 2). Monocytic pleocytosis in cerebrospinal fluid, May-Grünwald Giemsa x1000 (Cytospin) (**C**). Flow cytometry in CSF showed meningeal infiltration by 93% monocytes (red) and 7% lymphocytes (purple) (**D**). SSC = side scatter. (**E**) Cerebrospinal fluid, May-Grünwald Giemsa (MGG) stain, scale bar = 20 microns. Emperipolesis in a giant histiocytic cell: transcytoplasmic migration of two red blood cells (arrows)

A 65-year-old man presented in November 2021 with febrile anasarca, splenomegaly and pancytopenia (Table 1). Bone marrow aspiration revealed no central cause for the pancytopenia. PET-CT demonstrated infiltration of the adrenal glands and perirenal region, and osteo-medullary, pericardial, pleural, and peritoneal uptake. Adrenal gland biopsy confirmed ECD diagnosis, with the presence of the *MAP2K1 c.170 A > C*, p.(Lys57Thr) mutation (variant allele frequency: 31%) in histiocytes. In June 2022, the patient was hospitalized for ataxia and confusion. Brain MRI disclosed diffuse pachymeningitis (Fig. 1A-B) concomitant with leptomeningitis. Lumbar puncture revealed pleocytosis (50 cells/mm³, 75% monocytes). The monocytes had a dysmorphic appearance on pathological analysis. The patient was diagnosed with monocytic meningitis secondary to CNS infiltration in a context of ECD. A CSF liquid biopsy revealed a *MAP2K1 c.170 A > C* mutation (allele frequency: 44%). Another bone marrow aspiration showed an excess of monocytes (17%) with a phenotype consistent with type 1 chronic myelomonocytic leukemia (CMML), with evidence of *TET2*, *ASXL1*, *SRSF2* and *CBL* mutations. These mutations were also found in the CSF monocytes. Treatment with MEK-inhibitor binimetinib led to the resolution of monocytic meningitis within two weeks, accompanied by a quick neurological clinical and radiological improvement.

**Table 1:** Monocytic meningitis in histiocytosis (LCH, ECD, RDD, MH)

	Sex	Age	Diagnosis	Extra-neurological disease	Neurological symptoms	Mutation of BRAF/ MAPK pathway	Neurological imaging	Proteino-rrachia (mg/dL)	Glyco-rrachia (mg/dL)	CSF cells/mm³
Ueno et al., 2016	M	65	CNS-PMH	No	Impaired consciousness	ND	Subcortical masses, sulcus enhancements, leptomeningitis	175	8	59 (100% mono-histiocytes)
Nothem et al., 2021	F	42	CNS-PMH	No	Headache, cranial nerves palsy, ataxia, motor weakness	ND	Cranial nerve infiltration, pachymeningitis	146	< 10	518 (86% monocytes)
Polk et al., 2022	M	79	ECD	Yes	Confusion	ND	Ventriculitis (meningeal thickening on autopsy)	118	< 10	194 (87% monocytes)^b^
Actual series	M	65	ECD	Yes	Ataxia, confusion	MAP2K1	Pachymeningitis + leptomeningits	86	< 10	50 (75% monocytes)
Actual series	F	54	CNS-PMH	No	Ataxia	No identified	Leptomeningitis	445	ND	96 (60% monocytes)
Actual series	M	54	ECD/RDD	Yes	Tonico-clonic epilepsy, deafness	No identified	Leptomeningitis + nerve plexus infiltration	220	ND	70 (95% monocytes)

Abbreviations: CMML = chronic myelomonocytic leukemia; CNS-PMH = central nervous system primary malignant histiocytosis; ECD = Erdheim-Chester disease; LCH = Langerhans cell histiocytosis; MDS-LB = myelodysplastic syndrome with low blast; MH = malignant histiocytosis; ND = no data; RDD = Rosai-Dorfman disease; VAF = variant allele frequency

^a^ abstract available only

^b^ Initial lumbar puncture revealed neutrophilic pleocytosis. Monocytic pleocytosis was observed on day 12

### Case 2

In March 2021, a 54-year-old woman presented with recent-onset ataxia (Table 1). Cerebral MRI revealed leptomeningitis, and CSF analysis showed pleocytosis (96 cells/mm³, 60% monocytes). A diagnosis of neurosarcoidosis was established, leading to corticosteroids and cyclophosphamide treatments. In September 2021, the patient’s neurological condition worsened. CSF showed a significant infiltration of monocytes, and cytometry on CSF showed infiltration by 93% (Fig. 1C-D). Cerebral MRI demonstrated an exacerbation of the previously described lesions. A meningeal biopsy was performed, leading to a CNS primary malignant histiocytosis (CNS-PMH) diagnosis. No BRAF or MAPK pathway mutations were identified. MEK-inhibitor binimetinib was however introduced with a partial response. During this period of treatment, confirmation was obtained for a diagnosis of associated-myelodysplastic syndrome with a complex karyotype. Unfortunately, we observed neurological progression after two months of treatment. She received afterwards several lines of chemotherapy, with no response. She died in May 2022.

### Case 3

A 54-year-old man experienced epileptic seizure in March 2011 (Table 1). Initial investigations revealed pachymeningitis with lymphocytic meningitis. Despite an extensive diagnostic evaluation, no definitive diagnosis was reached. The patient developed progressive deafness in February 2013. MRI revealed a progression of pachymeningitis into the internal auditory meatus. A PET scan identified hypermetabolic foci in the proximal diaphysis of the right humerus and left femur, along with hypermetabolism in the brachial and lumbosacral nerve plexuses. A bone biopsy revealed the presence of an inflammatory histiocytic and lymphoplasmacytic infiltrate. A meningeal biopsy was performed, revealing the presence of a lymphoplasmacytic and histiocytic infiltrate with a preponderance of polyclonal plasmacytes. Despite the low IgG4/IgG ratio obtained on meningeal biopsy, a diagnosis of IgG4 disease was established. From 2013 to 2018, this patient received several successive treatments, including corticoids, rituximab, methotrexate, mycophenolate mofetil, azathioprine, and cyclophosphamide. In 2018, a peri-renal infiltration has been demonstrated. Biopsy results were suggestive of ECD. No *BRAF* mutation was detected. Lumbar puncture led to the detection of monocytic meningitis with emperipolesis (Fig. 1E). A revaluation of the 2013 meningeal biopsy resulted in the diagnosis of RDD. Ultimately, a diagnosis of mixed histiocytosis ECD/RDD with CNS involvement was established. The patient was treated with MEK-inhibitor cobimetinib, leading to clinical and radiological improvement until now.

## Discussion

Histiocytosis is the umbrella term given to a group of multisystem diseases characterized by the infiltration of histiocytes into several organs [1]. CNS involvement is detected in approximatively 5% of LCH patients, presenting as either tumoral manifestations (meningeal, choroidal, or parenchymal masses), or neurodegenerative syndrome [2]. CNS involvement is detected in 25 to 50% of ECD cases, manifesting as either tumoral (pachymeningitis or tumoral infiltration of the meninges) and neurodegenerative disease. The brainstem is frequently involved. The prevalent clinical manifestation is cerebellar syndrome [3], and its presence is linked to the *BRAF V600E* mutation [4]. Despite high frequency of meningeal involvement, CSF generally appears normal in neurological LCH and ECD [5]. Neurological RDD presents as dura-attached lesions resembling meningiomas [5]. Most diagnoses are established through the biopsy of meningeal or cerebral lesions.

We conducted a review of case reports focusing on CSF pleocytosis associated with histiocytosis. Meningitis was predominantly monocytic (> 50%) in three cases (Table 1), two of which involved CNS-PMH [6, 7]. The third case had ECD with neuromeningeal involvement [8]. The other cases (16 patients) described mixed pleocytosis (with monocytes or histiocytes) or exclusive lymphocytic pleocytosis [9–22]. The associated types of histiocytosis were MH, mixed LCH/ECD, LCH and RDD. The available clinical, radiological, and biological data are presented in Tables 1 and 2. Cranial nerve impairment is a prevalent feature, but confusion, altered consciousness, motor deficiencies, psychiatric disorders and bulbar damage are also common. Neurological manifestations commonly coincide with histiocytosis diagnosis but may appear later in some cases. Extra-neurological involvement was observed in all cases of histiocytosis except for CNS-PMH and RDD.

**Table 2:** Mixed/lymphocytic meningitis in histiocytosis (LCH, ECD, RDD, MH)

	Sex	Age	Diagnosis	Extra-neurological disease	Neurological symptoms	Mutation of BRAF/ MAPK pathway	Neurological imaging	Proteino-rrachia (mg/dL)	Glyco-rrachia (mg/dL)	CSF cells/mm³
Carbone et al., 1980	M	53	MH	Yes	Yes (ND)	ND	ND	ND	ND	Presence of histiocytes
Carbone et al., 1980	M	53	MH	Yes	No	ND	ND	ND	ND	Presence of histiocytes
Fujisawa et al.^a^, 1992	F	53	MH	Yes	Visual disturbance and lagophthalmos	ND	ND	ND	ND	Presence of histiocytes
Hiroshe et al., 1994	M	61	MH	Yes	Headache, VI palsy	ND	Ventricules/ fornix enhancement	720	9	Presence of histiocytes
Schroder et al.^a^, 1994	ND	ND	MH	ND	Psychiatric disorders	ND	Normal	ND	ND	Lymphocytes
Schroder et al.^a^, 1994	ND	ND	MH	ND	ND	ND	Normal	ND	ND	Lymphocytes
Stojkovic et al., 2000	F	18	Mixed LCH/ECD	Yes	Loss of vision, deafness	ND	Sinus, optic nerves, thalamus and meningeal infiltration	Normal	0.1	50 (presence of histiocytes)
Ghosal et al., 2001	M	28	LCH	Yes	Headache, limping gait, aphasia, hemiplegia, VII palsy	ND	Cystic left temporo-parietal lesions, cerebral peduncle	ND	ND	Mixed (presence of histiocytes)
Prosch et al., 2003	M	3	LCH	Yes	No	ND	Thickened pituitary stalk	ND	ND	94 (56% lymphocytes, 44% monocytes)
Colovic et al.^a^, 2007	F	64	MH	Yes	Headache, paraparesis, loss of consciousness	ND	Normal (computed tomography)	ND	ND	Presence of histiocytes
Kraeft et al., 2007	M	54	RDD	No	Back pain	ND	Pachymeningitis	ND	ND	Lymphocytes, histiocytes + emperipolesis
Nalini et al., 2012	H	35	RDD	No	Headache, blurred vision, hearing impairment	ND	Meningeal, optic nerves and suprasellar infiltration	92	85	480 (Lymphocytes)
Id Baih et al., 2014	F	40	MH	No	Right side paresthesia, neck pain, memory impairment	BRAF V600E	Left temporal masse	176	12	31 (Presence of histiocytes)
So et al., 2015	M	59	CNS-PMH	No	Right side paresia	ND	Multiple brain and spinal lesions	ND	ND	Presence of histiocytes
Duarte-Celada et al., 2020	F	35	LCH	Yes	Confusion, cognitive decline	ND	Hypothalamic lesion extended to pituitary stalk	ND	ND	7 (100% lymphocytes)
Tommasino et al., 2022	M	45	LCH	Yes	Headache, weakness, vocal cord paralysis, ataxia, dysphagia	ND	Leptomeningitis, nerve V infiltration	ND	ND	Mixed (presence of histiocytes)

Abbreviations: CMML = chronic myelomonocytic leukemia; CNS-PMH = central nervous system primary malignant histiocytosis; ECD = Erdheim-Chester disease; LCH = Langerhans cell histiocytosis; MDS-LB = myelodysplastic syndrome with low blast; MH = malignant histiocytosis; ND = no data; RDD = Rosai-Dorfman disease; VAF = variant allele frequency

^a^ abstract available only

^b^ Initial lumbar puncture revealed neutrophilic pleocytosis. Monocytic pleocytosis was observed on day 12

In our first patient, neurological progression occurred seven months after the initial diagnosis, and was marked by the emergence of pachymeningitis, leptomeningitis and monocytic pleocytosis. We identified the same *MAP2K1* mutation in the CSF monocytic population by liquid biopsy, confirming that this population was clonal rather than reactive. Pieri et al. already demonstrated the utility of liquid biopsy on CSF for detecting *BRAF*^V600E^ mutation in an ECD patient with neurological involvement [23]. Using an anti-MEK led to a rapid clinical and radiological neurological response, with meningeal infiltration disappearing. Our second patient highlights the diagnostic challenges associated with CNS-PMH. This is an exceptionally rare condition characterized by a poor prognosis [20]. Molecular analysis did not reveal any mutations in the MAPK pathway, and using an anti-MEK did not improve the clinical situation in this case. However, in the presence of a CNS-PMH, identifying a mutation in this pathway should prompt the consideration of targeted therapy [19]. The third patient was diagnosed with histiocytosis seven years after the onset of symptoms. Histiocytosis became apparent with the emergence of perirenal involvement and the confirmation of monocytic meningitis and signs of emperipolesis. Emperipolesis in CSF samples is rarely described in RDD, but its presence is highly suggestive of this disease [17]. In this case, despite the absence of a detectable mutation in the MAPK pathway, the anti-MEK treatment produced an excellent neurological response.

Given its rarity, the presence of monocytic meningitis should immediately prompt a suspicion of histiocytosis, particularly if accompanied by typical manifestations. Conversely, isolated neurological manifestations of histiocytosis may be more suggestive of CNS-PMH or RDD. In cases of neuromeningeal involvement, lumbar puncture and liquid biopsy can sometime overcome the need for a meningeal biopsy. The molecular characterization of histiocytosis is essential for considering the use of targeted therapy. However, the lack of an identified mutation should not preclude the use of anti-MEK therapy, as illustrated in our third case. Among our patients, two have shown a prolonged response to MEK inhibitors, with improvement in neurological damage and resolution of monocytic meningitis.

## Data Availability

No datasets were generated or analysed during the current study.

## Abbreviations

CMML: Chronic myelomonocytic leukemia
CSF: Cerebrospinal fluid
CNS: Central nervous system
CNS-PMH: Central nervous system primary malignant histiocytosis
ECD: Erdheim-Chester disease
LCH: Langerhans cell histiocytosis
MH: Malignant histiocytosis
MRI: Magnetic resonance imaging
PET: Positron emission tomography
RDD: Rosai-Dorfman disease
